# Neuroanatomical photogrammetric models using smartphones: a comparison of apps

**DOI:** 10.1007/s00701-024-06264-y

**Published:** 2024-09-24

**Authors:** Amedeo Piazza, Sergio Corvino, Daniel Ballesteros, Alice Campeggi, Edoardo Agosti, Simona Serioli, Francesco Corrivetti, Carlo Bortolotti, Matteo De Notaris

**Affiliations:** 1 Department of Neurosurgery, “Sapienza” University, Rome, Italy; 2 Laboratory of Neuroscience, EBRIS Foundation, Salerno, Italy; 3 Department of Neuroscience, Reproductive and Odontostomatological Sciences, Division of Neurosurgery Federico II, School of Medicine, University of Naples “Federico II”, 80131 Naples, Italy; 4 Neurosurgery Department, Instituto Nacional de Neurología y Neurocirugia, Manuel Velasco Suárez, Ciudad de Mexico, México; 5 Surgical and Medical Sciences and Translational Medicine, University of Rome “Sapienza”, Rome, Italy; 6 Division of Neurosurgery, Department of Medical and Surgical Specialties, Radiological Sciences and Public Health, University of Brescia, Brescia, Italy; 7 Division of Neurosurgery, Istituto Delle Scienze Neurologiche Di Bologna, IRCCS Bellaria Hospital, Bologna, Italy; 8 Unit of Neurosurgery, University Hospital San Giovanni Di Dio E Ruggi d’Aragona, University of Salerno, Fisciano, Italy

**Keywords:** 3D model, Photogrammetry, Pterional approach, Anatomy, Skull base, Training

## Abstract

**Objectives:**

A deep knowledge of the surgical anatomy of the target area is mandatory for a successful operative procedure. For this purpose, over the years, many teaching and learning methods have been described, from the most ancient cadaveric dissection to the most recent virtual reality, each with their respective pros and cons. Photogrammetry, an emergent technique, allows for the creation of three-dimensional (3D) models and reconstructions. Thanks to the spreading of photogrammetry nowadays it is possible to generate these models using professional software or even smartphone apps. This study aims to compare the neuroanatomical photogrammetric models generated by the two most utilized smartphone applications in this domain, Metascan and 3D-Scanner, through quantitative analysis.

**Methods:**

Two human head specimens (four sides) were examined. Anatomical dissection was segmented into five stages to systematically expose well-defined structures. After each stage, a photogrammetric model was generated using two prominent smartphone applications. These models were then subjected to both quantitative and qualitative analysis, with a specific focus on comparing the mesh density as a measure of model resolution and accuracy. Appropriate consent was obtained for the publication of the cadaver's image.

**Results:**

The quantitative analysis revealed that the models generated by Metascan app consistently demonstrated superior mesh density compared to those from 3D-Scanner, indicating a higher level of detail and potential for precise anatomical representation.

**Conclusion:**

Enabling depth perception, capturing high-quality images, offering flexibility in viewpoints: photogrammetry provides researchers with unprecedented opportunities to explore and understand the intricate and magnificent structure of the brain. However, it is of paramount importance to develop and apply rigorous quality control systems to ensure data integrity and reliability of findings in neurological research. This study has demonstrated the superiority of Metascan in processing photogrammetric models for neuroanatomical studies.

## Introduction

In every field of surgery, a deep knowledge of the surgical anatomy of a certain target area is mandatory for a successful operative procedure. For this purpose, over the years, many teaching and learning methods have been described, from the most ancient cadaveric dissection [17, 29] to the most recent virtual reality [31], each with respective limitations. Until the last decade, anatomy has always been studied on books and atlas, the main drawback being the bi-dimensional aspect of images and thus the inability to perceive the actual anatomical depth. To overcome this major limit, several technologies have been developed like 3D model DICOM Based [6], 3D photos [4], 3D printable models [19], among which photogrammetry [1, 7, 9, 10, 18, 19, 27, 28, 30].

The introduction of photogrammetry techniques is revolutionizing this field, offering powerful tools that overcome the limitations associated with 2D images, particularly in terms of depth perception and viewpoints.

Photogrammetry, a method based on the analysis of photographs, allows for the creation of three-dimensional (3D) models and reconstructions. Advanced software like Agisoft Metashape (Agisoft LLC, St. Petersburg, Russia), ReCap Pro (Autodesk, San Rafael, California, USA), Reality Capture Beta (Capturing Reality, Bratislava, Slovakia), [2, 3, 14, 23–25, 30], present the opportunity to generate highly accurate photogrammetric models. By capturing multiple images of a brain specimen from various angles and processing them through professional software, researchers can create detailed and precise 3D representations of the brain structure [28].

The use of professional software for photogrammetry in Neuroanatomy offers significant advantages in terms of accuracy. Sophisticated algorithms are employed to align images, calculate camera positions and generate highly detailed 3D models. However, managing these kinds of software requires a certain level of expertise and technical proficiency characterized by a gradual learning curve since the process of image capturing, marking control points and optimizing the model can be complex and time-consuming. Researchers must have a solid knowledge of the software and photogrammetry principles to achieve accurate results.

In addition to professional software, smartphone apps have also been developed as accessible tools for photogrammetry in Neuroanatomy studies [5, 21, 22, 24, 27, 30]. These apps leverage the built-in cameras of smartphones and offer user-friendly interfaces for capturing images and generating 3D models. Smartphone apps provide a convenient and straightforward solution at the expense of a certain level of accuracy compared to professional software, since they are built on simplified algorithms.

Smartphone apps are designed to be more user-friendly and accessible, allowing researchers to quickly capture images and generate basic 3D models without extensive technical knowledge. Smartphone apps may offer a valuable but less accurate alternative for researchers seeking quick and convenient 3D reconstructions of brain specimens, especially in the field of neurosurgery. These advancements in photogrammetry have broadened the horizons of neuroanatomical research, empowering researchers with diverse tools and approaches to further our understanding of the brain.

This study aims to compare, through quantitative analysis, the differences between the neuroanatomical photogrammetric models generated by two smartphone apps, namely Metascan (Abound Labs Inc., New York, NY, US), and 3D Scanner (Lanns lab, New York, NY, US).

These two applications have been widely used in previous research for generating photogrammetric human models [5, 8, 12, 20, 27], and among all the apps available, their free versions do not require any device other than a smartphone, which is why they were selected for this study.

## Materials and methods

### Specimens

Two head human specimens (4 sides) embalmed and injected with red and blue latex for arterial and venous blood vessels underwent a standard frontotemporal approach with the assistance of a 3D exoscope (Vitom, Karl Storz, Tuttlingen, Germany). The heads were fixed using a 3-pin head holder. Each step of the surgical procedure was separately investigated.

In accordance with Italian law and the policies of our institution, we hereby state that the use of specimens for academic research purposes within the scope of this study does not require formal approval from an ethics committee. Our practices comply with all relevant national regulations governing the ethical use of such specimens in a research setting.

### Dissection procedure

The anatomical dissection was divided into five steps to expose well-defined structures:

Step 1: skin.

Step 2: frontotemporal C-shaped skin incision and exposure of fronto-temporal fascia sparring the superficial temporal artery (STA).

Step 3: retrograde dissection according to Oikawa [15] of temporalis muscle to expose the pterional region underneath.

Step 4: frontotemporal craniotomy and dural opening to expose frontal and temporal lobes and opercula, with sylvian fissure.

Step 5: sylvian fissure splitting to expose the peri-chiasmatic region, in particular: middle cerebral artery, internal carotid artery, posterior communicating artery, anterior choroidal artery, ipsilateral anterior cerebral artery (segment A1), optic nerves, optic chiasm, ipsilateral oculomotor nerve.

### Photogrammetry and 3D model

Each described step of the dissection was scanned using the dual camera system of an iPhone 11 Pro (Apple Inc., CA, USA). A total of 120 photos were taken for each step while 10% of them were discarded being out of focus or out of field.

The selected photos of each step were separately processed using either Metascan (Abound Labs Inc., New York, NY, US) or 3D scanner app (Lanns lab, New York, NY, US) to create two 3D models for each step, set to the maximum possible resolution. Two selected smartphone apps were compared through quantitative and qualitative analyses, as well as in terms of app annual fee, model sharing, time for processing, user-friendly interface, the possibility of using manual focus and autofocus for each application.

Metascan and 3D Scanner are the most widely used applications in the field of photogrammetry. These programs do not require any additional device other than a smartphone, ending up being accessible to more researchers and not requiring extensive technical knowledge. [5, 8, 12, 27]

### Quantitative mesh analysis

Mesh analysis refers to the evaluation of a 3D mesh, which is the surface representation of an object or scene. A mesh consists of interconnected polygons that approximate the shape and structure of the subject being modeled. Mesh analysis involves examining various properties of the mesh, such as faces, vertices, edges, and face corners. It helps assess the quality, and density of the mesh and the representation of the captured subject.

Quantitative Mesh analysis was performed for each model generated by both smartphone apps using Blender (Blender Documentation Team. (2019). Blender 2.81 Reference Manual. https://docs.blender.org/manual/en/2.81/Blender Development Team. (2022). Blender (Version 3.1.0) [Computer software]. https://www.blender.org) following these parameters:

*Face:* A face is a single surface or polygon that makes up the mesh. Faces are often triangular or quadrilateral but can have more complex shapes like pentagons or hexagons. The number of faces in a mesh is a direct indicator of its density. A mesh with many faces has a higher level of details compared to one with fewer faces, which can impact the model resolution and overall complexity.

*Edge:* An edge is a line that connects two consecutive vertices of a face. Edge count can provide insights of the complexity of the mesh structure. A high number of edges may indicate a more detailed model, but it might also lead to increased computational complexity in some applications. Edges are critical in defining the boundaries of faces.

*Vertex:* A vertex is a 3D point that represents an intersection point between edges in the mesh. Vertices are crucial as they define the shape and positions of points in the mesh. The number of vertices is important for the accuracy of a mesh. A mesh with more vertices can reproduce complex curves and surfaces with greater precision, but this may also come with a higher demand for computational resources.

*Face Corner:* Face corners represent the points where the edges of a face meet. Despite not being frequently adopted as a parameter for definition, face corners count can be directly related to mesh complexity and detail resolution because they determine how the faces are connected to each other.

### Analyzed factors

Two smartphone apps were compared in terms of annual app fee, model sharing, time for processing, user-friendly interface, the possibility of using manual focus and autofocus for each application.

### Qualitative Visual inspection of the photogrammetric model

The 3D models were evaluated by an experienced neuroanatomist through visual inspection of specific structures at each step: step 1) representation of skin; step 2) representation of STA and muscle; step 3) representation of squamous suture, bone, and deep fascia of temporalis muscle; step 4) representation of cortical surface; step 5) representation of chiasm, optic nerve bilaterally, internal carotid artery, middle cerebral artery posterior communicating artery, anterior cerebral artery, anterior choroidal artery. The visual inspection was made using Blender (Documentation Team. (2019). Blender 2.81 Reference Manual https://docs.blender.org/manual/en/2.81/Blender Development Team. (2022). Blender (Version 3.1.0) [Computer software]. https://www.blender.org). The macroscopic differences between the models generated using the two apps were recorded using the snapshot function.

### Statistical analysis

Values were reported as mean ± standard deviation (SD). The ANOVA test was used to compare the quantitative continuous variables between Metascan and 3D Scanner among all steps. Statistical significance was predetermined at an alpha value of 0.05. (confidence interval 95%) BlueSky Statistics(Copyright © 2024 BlueSky Statistics) was used for data analysis (Table 1).

**Table:** Table 1

	Model step 1 (skin)			Model step 2 (superficial temporal fascia)			Model step 3 (bone deep fascia)			Model step 4 (cerebral parenchyma)			Model step 5 (final model)		
	Metascan *N*=4	3D scanner *N*=4	value *p*	Metascan *N*=4	3D scanner *N*=4	*p* value	Metascan *N*=4	3D scanner *N*=4	*P* value	Metascan *N*=4	3D scanner *N*=4	*P* value	Metascan *N*=4	3D scanner *N*=4	*P* value
Vertices Mean (SD).	50340 (231.084)	29780 (1457.418)	< 0.001	50330 (956)	25437 (873)	< 0.001	50518 (299.560)	28605 (1197.734)	< 0.001	50262 (986.445)	40194 (136.176)	< 0.001	64927 (120.161)	50484 (806.158)	< 0.001
Superiority of Metascan	69,06%			97,73%			76,59			25,04%			28,62		
Edges Mean (SD).	150337 (382.112)	88464 (1632.733)	< 0.001	150331 (116)	75437 (736)	< 0.001	150515 (2512.389)	84252 (1692.774)	< 0.001	150262 (2160.247)	119656 (1411.480)	< 0.001	194079 (4386.373)	150494 (2943.923)	< 0.001
Superiority of Metascan	69,97%			99,28%			78,58%			25,57%			28,96		
Faces Mean (SD).	99996 (1828.481)	58670 (1419.037)	< 0.001	100000 (2451)	50000 (2449)	< 0.001	99998 (3427.464)	55651 (1723.020)	< 0.001	99999 (1389.740)	79463 (1414.214)	< 0.001	129145 (5002.816)	99999 (1415.534)	< 0.001
Superiority of Metascan	70,46%			100%			79,76%			25,83			29,15		
Face corners Mean (SD).	299988 (5485.442)	176010 (4257.112)	< 0.001	300000 (7353)	150000 (7348)	< 0.001	299994 (10282.393)	166953 (5169.061)	< 0.001	299997 (4169.220)	238389 (4242.641)	< 0.001	387435 (15008.448)	299997 (4246.603)	< 0.001
Superiority of Metasca	70,43%			100%			79,69%			25,85%			29,15%		

## Results

### Quantitative mesh analysis (Table 1)

For each step, 4 models were generated, using both Metascan and 3D Scanner, for a total number of 40 photogrammetric models.

### Number of vertices

In the models processed with Metascan, on average, a superiority of 69.06% was observed in step 1, 97.73% in step 2, 76.59% in step 3, 25.04% in step 4, and 28.62% in step 5 compared to model processed with 3D scanner.

### Number of edges

In the model processed with Metascan, on average, a superiority of 69.97% was observed in step 1, 99,28% in step 2, 78.58% in step 3, 25.57% in step 4, and 28,96% in step 5 compared to model processed with 3D scanner.

### Number of faces

In the model processed with Metascan, on average, a superiority of 70,46% was observed in step 1, 100% in step 2, 79.76% in step 3, 25.83% in step 4, and 29.15% in step 5 compared to model processed with 3D scanner.

### Number of face corners

In the model processed with Metascan, on average, a superiority of 70,43% was observed in step 1, 100% in step 2, 79.69% in step 3, 25.85% in step 4, and 29.15% in step 5 compared to model processed with 3D scanner.

### App evaluation

The two applications taken into account present some comparable characteristics such as the processing time and the possibility of using cloud processing. 3D Scanner App offers also the possibility to process the model using a personal computer. Either with Metascan or 3D Scanner App it is possible to export the models in several formats or even export them in the form of short videos; it is even possible to edit the model with basic functions, such as *crop function*. Despite.

having both a user-friendly interface, 3D Scanner App adds also a brief description of each file extension option in the sharing section which is helpful for novices. Regarding image capture each one of them offers the possibility to capture the images with autofocus, but in 3D Scanner App it is also possible to capture images by video recordings *(auto capture function).* Despite this interesting function that reduces shooting time, the photos are burdened by a lack of definition. Metascan offers the possibility to adjust the focus manually for every single photo which helps capturing specific target regions located more deeply with respect to the surrounding structures.

The results of the evaluation of the two smartphone apps are summarized in Table 2.

**Table 2:** Apps evaluation

Price	Metascan	3D-Scanner
	6,99 $ monthly	Free, 10 model/month
Processing Time	25 min	20 min
User friendly interface	+	+
Model sharing	+	+
Cloud processing	+	+
Desktop processing	-	+
Edit mode	+	+
Autofocus	+	+
Manual focus	+	-
Capture from video	-	+

### Visual inspection of the photogrammetric model

**Fig. 1:**
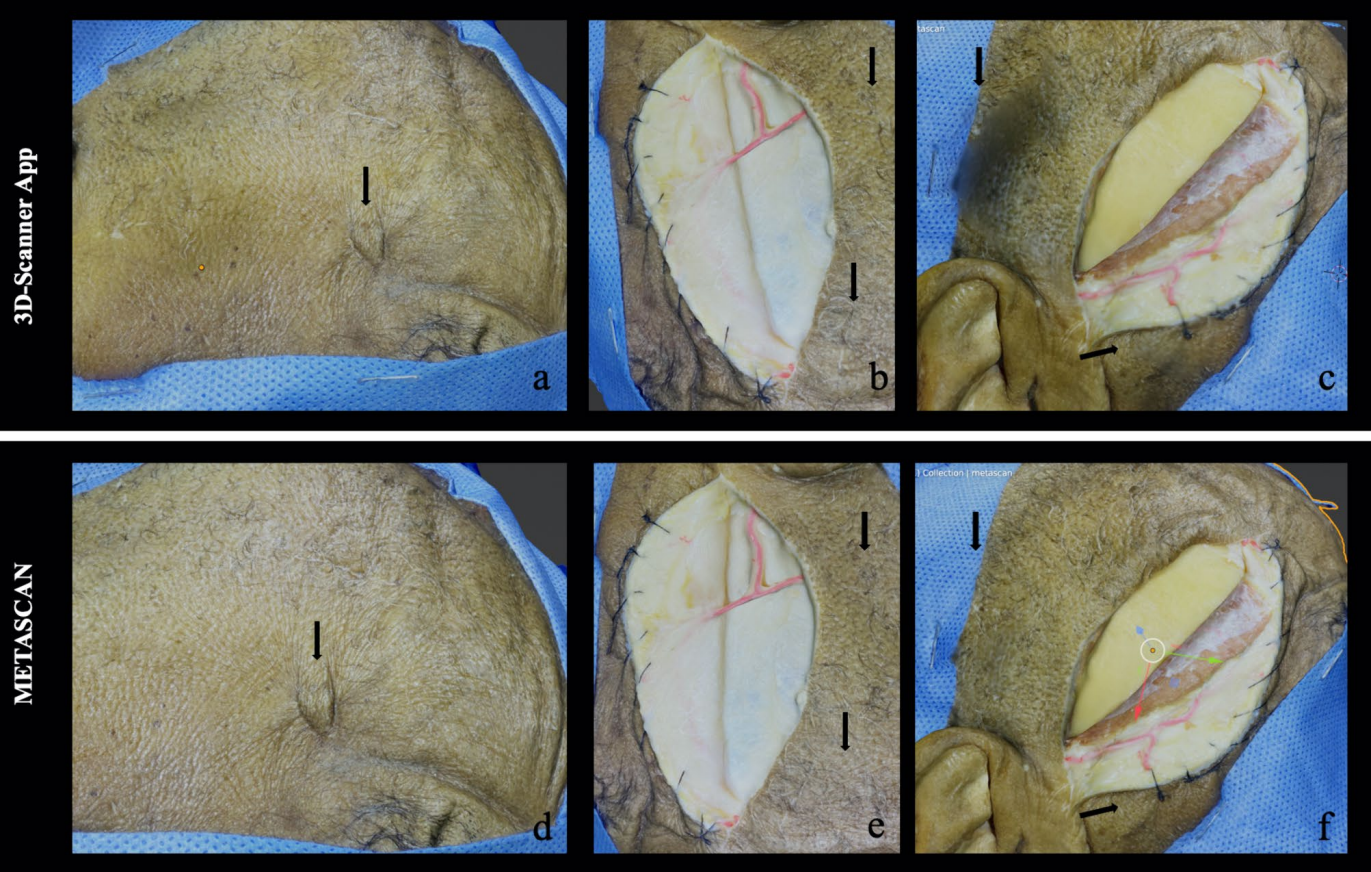
The photogrammetric model of Step 1 (letters a, d), Step 2 (letters b, e), and Step 3 (letters c, f) are presented. The first row shows the 3D model generated with Metascan while the lower one the models generated with 3D Scanner App. The black arrow indicates the major differences between each model at comparison

**Fig. 2:**
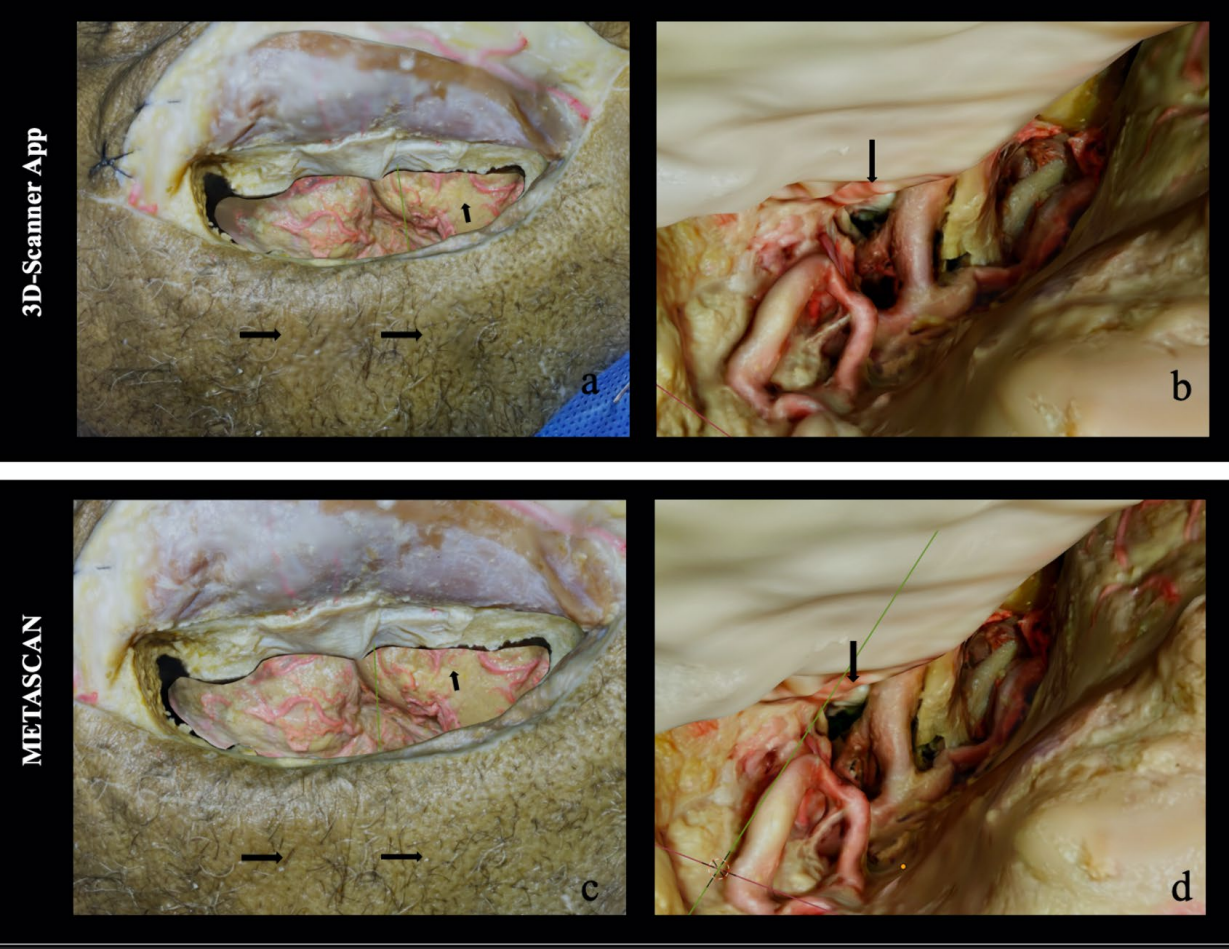
The photogrammetric model of Step 4 (letters a, c) and Step 5 (letters b, d) are presented. The first row shows the 3D model generated with Metascan while the lower one the models generated with 3D Scanner. The black arrow indicates the major differences between each model compared

Despite the significant difference of mesh density found among the models, in *Model 1* the visual inspection of these models seems similar except for a small skin imperfection just over a scar (Fig. 1 a, d). The complexity of the model increased in the subsequent steps and the differences at the visual inspection between the models generated by Metascan and 3D Scanner App became more evident. In Fig. 1 (b, e) the skin anterior to the frontotemporal incision appears out of focus, while in Fig. 1 (c, f) it is possible to see a significant image distortion in parieto-occipital region and along the skin lap. In Fig. 2 (a, c) some small cortical vessels are missing, and there are two areas of image distortion along the skin incision in the model generated using 3D scanner. Finally, in Fig. 2 (b, d), Posterior communicating artery (PcomA), Anterior choroidal artery (AchA), and their perforating branches are missing. Another important factor encountered during the visual inspection of the two models was the different zoom power that was greater in the models generated by Metascan.

The results of visual inspection are listed in Figs. 1 and 2.

## Discussion

The reconstruction of 3D anatomical models represents an innovative approach for anatomy education and learning, as interactive photogrammetric models can be navigated and visualized in augmented, virtual, or mixed-reality. Scientific evidence supports the use of these tools as an efficient path for anatomical understanding compared to traditional methods [11, 13]. Moreover, advancements in cloud-based processing have significantly reduced the computational resources required, facilitating the proliferation of smartphone applications created for this purpose. While numerous studies have been conducted on neuroanatomical photogrammetric models [3, 10, 12, 16, 25, 27, 28, 30], there remains a lack of standardized quality control methods. In this study, we aimed to address this limitation by evaluating the quality of models through the analysis of mesh density, a quantifiable parameter commonly used for assessment. Our findings revealed that photogrammetric models generated using Metascan exhibited superior mesh density compared to those produced with 3D Scanner in each step. Additionally, visual inspection of the photogrammetric model confirmed the superiority of Metascan, as highlighted by a higher occurrence of digital artifacts, a term commonly used in photography to denote a loss of definition, particularly in soft tissue representation during initial steps of the pterional approach and in the graphic depiction of the parasellar region, where the rendering of structures such as the posterior communicating artery (Pcom), anterior communicating artery (AcoA), and their perforating branches was not achieved satisfactorily.

By analyzing the percentage differences in mesh density between the two apps for each step, a reduction in Metascan's percentage superiority was observed, decreasing from 69% in step 1 to 28% in step 5 (Table 1). However, no reduction in the digital artifacts found in the models was observed. This may be due to the increase in surface area, depth of the dissection, and complexity of the model to be reconstructed in the subsequent steps. The value of mesh density found through Metascan has demonstrated to be enough for an accurate qualitative reproduction of neuroanatomical images.

Moreover, achieving comprehensive coverage with overlapped photographs within a field poses a challenge, particularly in light of the non-linear nature of neuroanatomy. The cerebral surface, characterized by sulci, fissures, gyri, and convolutions, presents a complex topography. Similarly, the basicranium, neurovascular structures contained within cisternal spaces, and bony features such as foramina, ridges, and osseous canals contribute to the intricate three-dimensional landscape.

Consequently, the development of tools capable of delivering high-fidelity reproductions of dissections is crucial.

As previously described, the use of photogrammetry in Neuroanatomy offers numerous advantages, to the extent that it could potentially be supplanting traditional two-dimensional images in the future.

However, it is essential to address the need for adequate quality control systems to ensure the accuracy and reliability of photogrammetric data. Errors in image registration can lead to erroneous conclusions and have significant implications in neuroanatomical research. For example, using the 3D model with numerous digital artifacts for measurements in neuroanatomical studies could affect the accuracy of the results. Therefore, method validation and the implementation of rigorous quality controls are essential to ensure scientifically valid results.

## Limitations

A major limitation is represented by the comparison of only two among the various smartphone apps available. The choice of comparing these two apps was based on their application in the field of human anatomy [8, 12, 26, 27], since on one hand they are the most cited in the literature up to now and, on the other hand, their free version does not require any adjunctive tools, unlike other apps [10].

## Conclusion

By enabling depth perception, capturing high-quality images and offering flexibility of viewpoints, photogrammetry provides researchers with unprecedented opportunities to explore and understand the intricate and magnificent structure of the brain. However, it is of paramount importance to develop and apply rigorous quality control systems to ensure data integrity and the reliability of findings for Neurological research.

In particular, this study demonstrates the superiority of Metascan when it comes to processing photogrammetric models for neuroanatomical studies.

Further studies should explore the availability of other quality control systems and evaluate the accuracy of linear measurements via photogrammetry.

## Data Availability

Data of the current original research are available from the corresponding author on reasonable request.

## Abbreviations

3D: Three-dimensional
2D: Two-dimensional
AR: Augmented reality
VR: Virtual reality
MRI: Magnetic Resonance Imaging
ROI: Region of interest
DICOM: Digital Imaging and Communications in Medicine
PcomA: Posterior communicating artery
AchA: Anterior choroidal artery
